# Evaluation of Antioxidative and Neuroprotective Activities of Total Flavonoids From Sea Buckthorn (*Hippophae rhamnoides* L.)

**DOI:** 10.3389/fnut.2022.861097

**Published:** 2022-06-21

**Authors:** Zheng Wang, Wenqian Wang, Changlong Zhu, Xiangdong Gao, Weihua Chu

**Affiliations:** State Key Laboratory of Natural Medicines, School of Life Sciences and Technology, China Pharmaceutical University, Nanjing, China

**Keywords:** *Hippophae rhamnoides* L., flavonoids, antioxidative activity, neuroprotective activity, *C. elegans*

## Abstract

The aim of this study was to investigate the antioxidative and neuroprotective activities of total flavonoids from sea buckthorn (*Hippophae rhamnoides* L.) (TFH). Results indicated that TFH possessed DPPH radicals, hydroxyl radicals and superoxide anions scavenging activities. The neuroprotective potential was assessed with acetylcholinesterase (AChE) and monoamine oxidase A (MAO-A). The inhibition rates of AChE and MAO-A by 50 μg/ml TFH were 75.85 and 51.22%, respectively. The *in vivo* antioxidative and neuroprotective potential of TFH were explored in *Caenorhabditis elegans*. In the longevity assay, TFH (50 μg/ml) significantly increased the lifespan of wild-type *C. elegans* (29.40%). In the hydrogen peroxide-induced oxidative stress challenge, the antioxidant capacity of TFH-treated wild-type *C. elegans* was significantly enhanced. The *C. elegans* mutant strain CL4176 was used to study the neuroprotective effect of TFH *in vivo*. Results showed that TFH significantly delayed paralysis in *C. elegans* CL4176. Our study suggested total flavonoids from sea buckthorn (*Hippophae rhamnoides* L.) had the potential as an antioxidative and neuroprotective agent to extend aging and treat neurodegenerative diseases.

## Introduction

With the lifestyle changes and the aging of the population in modern society, diseases such as neurodegenerative disorders pose a serious threat to human health. Aging is a major risk factor for most neurodegenerative diseases, including Alzheimer’s disease (AD) and Parkinson’s disease (PD) (1). Biochemical changes in brain are responsible of age-related cognitive impairment and the development of these neurodegenerative diseases. Alzheimer’s disease is currently treated primarily with acetylcholinesterase (AChE) inhibitors, which can increase the concentration of acetylcholine in the brain, thus ameliorating the cognitive and mental decline associated with this neurodegenerative disease (2). Recent studies have also shown that Monoamine oxidase A (MAO-A) has the potential to play a role in the disease-modifying treatment of neurodegenerative diseases (3). In addition, one of the main causes of aging and age-related diseases is the production of reactive oxygen species (ROS) during the metabolic process (4, 5). Therefore, there is an increasing need to find compounds with antioxidant and neuroprotective effects to delay aging and prevent or treat aging-related diseases.

In the recent years, there has been a dramatic increase in usage of plant-derived foods in preventive diseases area. Polyphenolic compounds, especially flavonoids, are known as most common phytochemicals which possess a multiple range of biological effects. Sea buckthorn (*Hippophae rhamnoides* L.) is a medicinal and food plant which belongs to the Elaeagnaceae family and which has attracted much research attention because of its, rich in nutrients and bioactive substances (6). The chemical composition of sea buckthorn is mainly flavonoids and saponins, and it is also rich in vitamins, amino acids, trace elements and other nutrients which possess a variety of biological activities and health effects such as antioxidant, anti-tumor, hypoglycemic, improve blood lipid metabolism and other physiological effects (7).

The aim of this study was to investigate the antioxidative activity of total flavonoids from sea buckthorn (*Hippophae rhamnoides* L.) (TFH), and we also further evaluate its neuroprotective activity using *Caenorhabditis elegans* (*C. elegans*) as an *in vivo* model. These findings highlighted those total flavonoids from sea buckthorn (*Hippophae rhamnoides* L.) had the potential to delay aging and prevent or treat neurodegenerative diseases associated with aging.

## Materials and Methods

### Reagents and Chemicals

Total flavonoids from sea buckthorn (*Hippophae rhamnoides* L.) (TFH) was purchased from Nanjing Yuanzhi Biotechnology Co., Ltd. (Nanjing, China) which was extracted from the fruits. The main components of TFH is isorhamnetin, and the purity is more than 95%. The concentration of TFH solution [dissolved in Dimethyl sulfoxide (DMSO)] used in this study was 10, 20, and 50 μg/ml (8, 9). Other chemical regents used in this study were obtained from Sigma-Aldrich Merck (Sigma-Aldrich, St. Louis, MO, United States).

### *Caenorhabditis elegans* Strains and Maintenance

The *C. elegans* strains used in this study were N2 Bristol (wild-type) and CL4176 [*smg-1(CC546) I; dv*] *s27X*. The wild-type strain was obtained from Caenorhabditis Genetics Center (University of Minnesota, United States). The mutant strain was kindly provided by Prof. Hongbing Wang (School of Life Sciences and Technology, Tongji University, Shanghai, China). The wild-type *C. elegans* strain was grown at 20°C (CL4176 at 16°C) on nematode growth medium (NGM) agar plates seeded with the *E. coli* strain OP50 under standard laboratory conditions (10).

### *In vitro* Antioxidant Activity

#### DPPH Assay

DPPH-free radicals scavenging activity of the different concentrations of TFH was determined as described by Shi et al. (11). First, TFH solution (different concentrations) was filter-sterilized (0.22 μm). Then, 0.4 ml of TFH solution was mixed with 1.6 ml freshly prepared DPPH solution (0.2 mM in ethanol) and allowed to react for 25 min in dark. The control group contained the corresponding volume of DMSO and DPPH of TFH. Then, the absorbance was measured at 517 nm. The value of OD_517_ of experimental group and control group was recorded, respectively, as A_1_ and A_0_. The scavenging rate is expressed as follows:


$$\mathrm{Scavenging}\mathrm{rate}=\left(A_{0}-A_{1}\right)/A_{0}\times 100\%$$

#### Hydroxyl Radical Assay

The hydroxyl radical scavenging ability was determined according to the method by Yu et al. with some modifications (12). First, 1.4 ml of sodium salicylate (5 mM), FeSO_4_ (5 mM), and H_2_O_2_ (3 mM) were mixed with 100 μl of TFH solution or DMSO (control group contained the corresponding volume of DMSO of TFH). Then, the mixture was incubated at room temperature for 50 min. The absorbance of the solution was measured at 510 nm. The value of OD_510_ of experimental group and control group was recorded, respectively, as A_1_ and A_0_. The scavenging rate is expressed as follows:


$$\mathrm{Scavenging}\mathrm{rate}=\left(A_{0}-A_{1}\right)/A_{0}\times 100\%$$

#### Superoxide Anion Assay

The ability to scavenge superoxide anion was tested by the method as described by Shivangi et al. (13) with some modifications. The reaction mixture contained 1 ml TFH solution or DMSO (control group), 2.8 ml Tris–HCl buffer (0.05 M, pH 8.2) and 0.1 ml pyrogallic acid (0.05 M). Then the mixture was reacted for 4 min at room temperature in dark. 80 μl of 0.1 M HCl was added to stop the reaction and absorbance was measured at 325 nm. The value of OD_325_ of experimental group and control group was recorded, respectively, as A_1_ and A_0_. The scavenging rate is expressed as follows:


$$\mathrm{Scavenging}\mathrm{rate}=\left(A_{0}-A_{1}\right)/A_{0}\times 100\%$$

### Neuroprotective Potential

#### Acetylcholinesterase Enzyme Inhibitory Activity

Inhibitory AChE activity was detected using commercial acetylcholinesterase assay kit (Nanjing Jiancheng Bioengineering Institute, Nanjing, China) according to the manufacturer’s instruction (14). All experiments were performed in triplicate.

#### Monoamine Oxidase A Enzyme Inhibitory Activity

The inhibition of MAO-A activity was assessed using commercial MAO monoamine oxidase assay kit (Nanjing Jiancheng Bioengineering Institute, Nanjing, China) according to the manufacturer’s instruction (15). All experiments were performed in triplicate.

### *Caenorhabditis elegans* Assays

#### Lifespan Assay

This assay has been carried out following the method by Sugawara et al. (16) with a slight modification. First, 100 μl of fresh overnight culture of *E. coli* OP50 was, respectively, spread on NGM plates containing different concentrations of isorhamnetin (treatment group) or DMSO (control group). 5-fluorodeoxyuridine (final concentration of 70 mmol/l) was added to prevent nematodes from hatching. Synchronized wild-type *C. elegans* worms (L4 stage) were transferred to above NGM plates and cultured at 20°C. The number of live worms was counted every 24 h. A worm was considered dead when it failed to respond to gentle touch with a worm picker. The experiment was done in triplicate. The mean lifespan was estimated as follows:


$$MLS=\frac{1}{N}\sum_{j}\frac{x_{j}+x_{j+1}}{2}d_{j}$$

Where *j* is the age (day), *d*_*j*_ is the number of worms that died in the age interval (*x*_*j*_, *x*_*j*+1_), and *N* is the total number of worms.

#### Assessment of Resistance to Oxidative Stress

The antioxidant activity of TFH on *C. elegans* (N2) was assessed based on previous method with slight modifications (17). First, 100 μl of fresh overnight culture of *E. coli* OP50 was, respectively, spread on NGM plates containing different concentrations of TFH (treatment group) or DMSO (control group). 5-fluorodeoxyuridine (final concentration of 70 mmol/l) was added to prevent nematodes from hatching. The synchronized worms (L4 stage) were transferred to above plates and cultured at 20°C for 3 days. After that, each group of 20 worms were transferred to an NGM plate containing 50 mmol/l hydrogen peroxide. The dead worms were counted after 16 h. Experiments were done in triplicate.

#### Paralysis Assay

The *C. elegans* strain CL4176 is a mutant strain that contains a temperature-sensitive mutation. The mutant expresses human amyloid β (1-42). The Aβ (β-Amyloid) peptide is expressed and aggregates in muscle cells, resulting in paralysis in the mutants. CL4176 worms were egg-synchronized onto fresh NGM plates with different concentrations of TFH (treatment group) or DMSO (control group) at 16°C (18). When the worms grew to L3 larvae, the temperature was raised to 25°C to induce Aβ transgene expression. After 36 h (recorded as 0 h), paralysis was scored at 16 h. Each assay was done in triplicate.

### Statistical Analysis

The experiments were repeated three times. Data analysis and mapping using GraphPad 8.3 software. Statistical differences between groups were determined using one-way ANOVA. The level of *p* < 0.05 was considered to be statistically significant, and the level of *p* < 0.01 was considered to be statistically extremely significant.

## Results

### Antioxidant Activities of Total Flavonoids From Sea Buckthorn (*Hippophae rhamnoides* L.)

#### DPPH-Free Radicals

**FIGURE 1:**
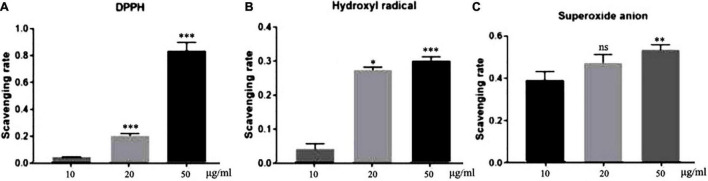
*In vitro* antioxidant potential of TFH. Results are represented as mean ± SEM of three independent replicates. ns, no significance; ***p < 0.001, **p < 0.01, *p < 0.05 vs. the 10 μg/ml group.

Elimination of DPPH-free radicals is the base of the commonly used antioxidant assays (19). As shown in Figure 1A, all three concentrations of TFH had a certain scavenging activity against DPPH-free radicals. Moreover, the scavenging activity increased with TFH concentration. Among them, the scavenging activity was highest at a concentration of 50 μg/ml, with a scavenging rate of 83.59%.

#### Hydroxyl Radicals

Hydroxyl radicals play an important role in the endogenous damage of DNA (20). As shown in Figure 1B, the scavenging rate of TFH was 30.07 and 27.45% at the concentrations of 50 μg/ml and 20 μg/ml, respectively. The scavenging rate was 4.27% at 10 μg/ml.

#### Superoxide Anions

The superoxide anion can induce oxidative stress and thus cause damage to biological molecules (21). As shown in Figure 1C, the highest clearance rate was 54.15% at a concentration of 50 μg/ml. The difference was that as the concentration decreased, the clearance rate appeared to decrease.

### Potential Neuroprotective Effect of Total Flavonoids From Sea Buckthorn (*Hippophae rhamnoides* L.)

The *in vitro* neuroprotective potential of TFH was determined through the AChE and MAO-A inhibitory activity, which are associated with neurodegenerative disorders (22). Results are summarized in Figure 3. All concentrations of TFH showed inhibitory activity against both AChE (Figure 3A) and MAO-A (Figure 3B), among them, the strongest inhibitory activity of TFH was observed at 50 μg/ml. When the concentration of TFH was 50 μg/ml, the AChE and MAO-A inhibitory rate were 75.85 and 51.22%, respectively. Results showed that TFH had potential neuroprotective effect.

**FIGURE 2:**
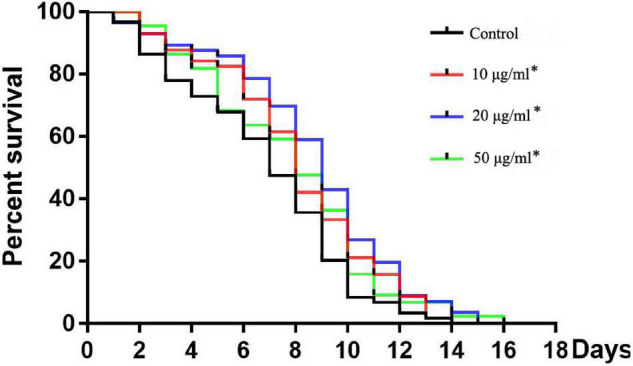
Effects of TFH on lifespan of *C. elegans*. Three replicates were used per treatment. Differences in survival curves between treatment and control groups were found in all TFH treated groups. Differences compared to control group were considered significant at *p ≤ 0.05.

**FIGURE 3:**
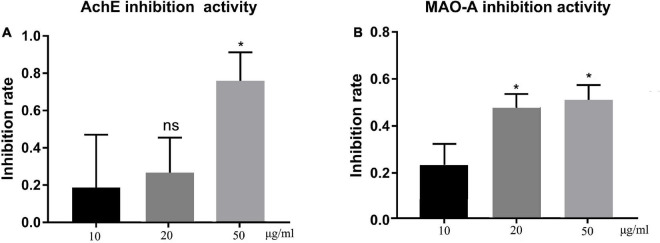
*In vitro* AChE and MAO-A inhibitory activity of TFH. Results are represented as mean ± SEM of three independent replicates. ns, no significance; *p < 0.05 vs. the 10 μg/ml group.

### Total Flavonoids From Sea Buckthorn (*Hippophae rhamnoides* L.) Enhances the Lifespan of *C. elegans*

All concentrations of TFH significantly (*p* < 0.05) increased the mean lifespan (MLS) of *C. elegans* (N2) compared with the control group (shown in Figure 2 and Table 1). In addition, the survival rate of all TFH-treated groups was higher than that of the control group during the whole experiment. Among the three concentrations, TFH at a concentration of 50 μg/ml is the most effective, increasing the MLS by 29.40%, followed by TFH at a concentration of 20 μg/ml by 21.86%, and TFH at a concentration of 10 μg/ml by 18.55%. Therefore, TFH had a life-prolonging effect on *C. elegans*.

**TABLE 1:** Effects of TFH on *C. elegans* (N2) longevity.

Concentration (μg/ml)	Maximum lifespan (days)	Median lifespan (days)	Mean ± SE (days)	Change (%)
0	14	7	6.36 ± 0.58	/
10	14	8	7.54 ± 0.17	+18.55
20	15	8	7.75 ± 0.13	+21.86
50	16	9	8.23 ± 0.31	+29.40

*Each value is expressed as mean ± SD. Experiments were done in triplicate.*

### Total Flavonoids From Sea Buckthorn (*Hippophae rhamnoides* L.) Enhances the Oxidative Stress Tolerance of *C. elegans*

**FIGURE 4:**
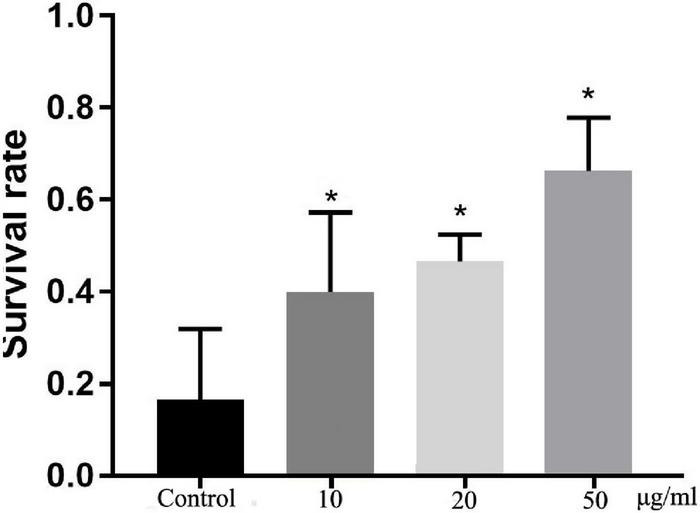
Effects of TFH on oxidative stress tolerance of *C. elegans*. Three replicates were used per treatment. Differences compared to control group were considered significant at *p ≤ 0.05.

Oxidative stress is harmful to life processes and especially responsible for aging and age-related diseases (23). After exposure to hydrogen peroxide, which is a known source of active oxygen, the survival rates of TFH-treated worms were significantly (*p* < 0.05) different with control group (shown in Figure 4). Among them, TFH had a maximum effect at a concentration of 50 μg/ml, and the survival rate of worms can achieve 66.67%. Hence, TFH improved tolerance to oxidative stress in *C. elegans*.

### Total Flavonoids From Sea Buckthorn (*Hippophae rhamnoides* L.) Delays the Paralysis of β-Amyloid Transgenic *C. elegans*

**FIGURE 5:**
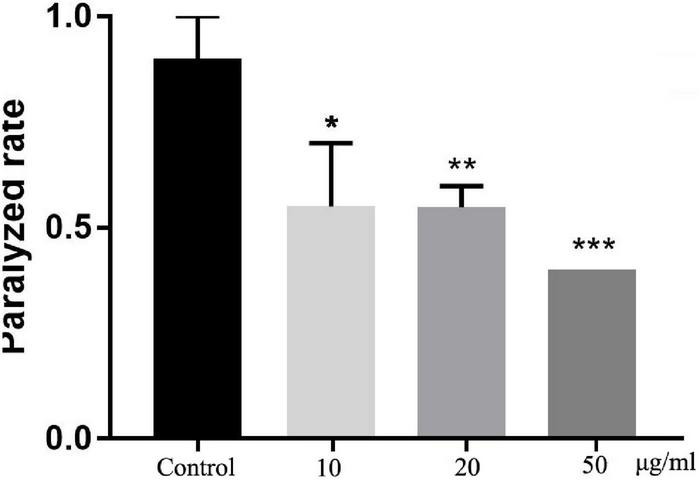
Effect of TFH on Aβ-induced paralysis in transgenic *C. elegans* CL4176. Three replicates were examined per treatment. Differences compared to control group were considered significant at *p ≤ 0.05, **p ≤ 0.01, and ***p ≤ 0.001.

To detect the neuroprotective effect of TFH *in vivo*, we fed different concentrations of TFH to transgenic *C. elegans* CL4176 strain and compared the paralysis rate at 16 h. Compared with the control group, the paralysis rate of worms in TFH-treated group was significantly reduced (shown in Figure 5). Among them, TFH had a maximum effect at a concentration of 50 μg/ml, and the paralysis rate was 40.00%. Our results showed that T FH exhibited significant delay of Aβ-induced paralysis.

## Discussion

In recent years, more and more plant-derived drugs have been used to prevent and treat diseases. There are approximately 4,000 flavonoids in plants, many of which have a wide range of biological activities (24). Sea buckthorn has a high content of flavonoids, which are widely present in the fruits, seeds and leaves, mainly including isorhamnetin, quercetin, kaempferol, prunetin, rutin, catechin and other flavonoid glycosides and their glycosides (such as isorhamnetin-3-O-β-D-glucoside, quercetin-3-O-β-D-glucoside, and kaempferol-3-O-rutinoside-7-O-rhamnoside, etc.) (25). In the current study, we investigated the antioxidant activity and neuroprotective effects of total flavonoids from sea buckthorn (*Hippophae rhamnoides* L.) (TFH). In the *in vitro* antioxidant assay, three concentrations (10, 20, and 50 μg/ml) of TFH were shown to scavenge DPPH-free radicals, hydroxyl radicals and superoxide anions. The scavenging rate of DPPH radicals, hydroxyl radicals and superoxide anions tended to increase as the concentration of TFH increased. According to previous report, flavonoids exhibited antioxidant activity. Isorhamnetin, one of the main compounds from Sea buckthorn could scavenge DPPH-free radicals and ABTS radicals in vitro and exhibited antioxidant activity (26, 27). Quercetin, as a flavonoid, has also been reported to have free radical scavenging properties (28). Polyphenolic compounds extracted from three Romanian Lamiaceae plants, sage (*Salvia officinalis*), rosemary (*Rosmarinus officinalis*) and lavender (*Lavandula angustifolia*) showed antioxidant activity (29). Further, in our study, all concentrations of TFH inhibited acetylcholinesterase and monoamine oxidase A *in vitro*. The strongest inhibitory activity of TFH was observed at 50 μg/ml. Similarly, isorhamnetin has previously been reported to inhibit AChE (30). Other flavonoids have also been reported to possess this activity, for example, 15 flavonoids isolated from *Eupatorium adenophorum* have been reported to have inhibitory activity against AChE (31).

This study also used *C. elegans* as a model to investigate the activity of TFH *in vivo*. The results showed that TFH prolonged the lifespan of wild-type *C. elegans*. In particular, TFH at 50 μg/ml increased the lifespan by 29.40%. It had been reported that the flavonoids in peony petals had anti-aging and anti-oxidative stress properties, including prolonging the lifespan and providing antioxidant activity (32). Similarly, the rutin had been reported to increase lifespan in *C. elegans* (33). In addition, our results showed that TFH enhanced the tolerance of *C. elegans* to oxidative stress induced by hydrogen peroxide. It had been reported that flavonoids kaempferol and fisetin can increase the survival of *C. elegans*, diminish the extent of induced oxidative stress in *C. elegans* (34). It is also found that isorhamnetin enhances cellular antioxidant defense against H_2_O_2_-induced cytotoxicity in C2C12 cells (35). Isorhamnetin also had a potential protective effect against oxidative stress in human RPE cells (36). So, it is suggested that the antioxidant activity of TFH is based on isorhamnetin.

The present study showed that TFH significantly reduced the paralysis rate in transgenic *C. elegans* CL4176 strain. At the 16 h after induction of paralysis, the paralysis rate was 90% in the control group, whereas the paralysis rate in the TFH-treated group (50 μg/ml) were 40.00%. These results suggested that TFH had neuroprotective effects. Flavonoids are well-documented for neuroprotective and neurorestorative effects against various neurodegenerative diseases (37, 38). It has been reported that flavonoids from Good King Henry (*Chenopodium bonus-henricus* L.) showed statistically significant neuroprotective activities on isolated rat brain synaptosomes using 6-hydroxydopamine *in vitro* model (39). Likewise, Agathisflavone, a flavonoid derived from *Poincianella pyramidalis* (Tul.), enhances the neuroprotective properties of microglia and astrocytes to significantly ameliorate glutamate-mediated neurotoxicity (40). However, the molecular mechanism and which compounds in TFH exerts the antioxidant and neuroprotective effects need to be further explored.

## Conclusion

In conclusion, the present study demonstrated that total flavonoids from sea buckthorn (*Hippophae rhamnoides* L.) had antioxidant activity and potential neuroprotective effects *in vitro* and *in vivo*. Furthermore, *C. elegans* can be used in aging and aging-related neurodegenerative disease studies. This study supported the potential application of total flavonoids from sea buckthorn (*Hippophae rhamnoides* L.) as an antioxidative and neuroprotective agent to delay aging and treat aging-related diseases in humans.

## Data Availability Statement

The original contributions presented in the study are included in the article/supplementary material, further inquiries can be directed to the corresponding authors.

## Author Contributions

WW: investigation, visualization, and writing – original draft. ZW and CZ: investigation, visualization, and methodology. WC and XG: supervision and writing – review and editing. All authors contributed to the article and approved the submitted version.

## Conflict of Interest

The authors declare that the research was conducted in the absence of any commercial or financial relationships that could be construed as a potential conflict of interest.

## Publisher’s Note

All claims expressed in this article are solely those of the authors and do not necessarily represent those of their affiliated organizations, or those of the publisher, the editors and the reviewers. Any product that may be evaluated in this article, or claim that may be made by its manufacturer, is not guaranteed or endorsed by the publisher.
